# Mechanical stress, metabolic reprogramming and stromal remodeling: an emerging paradigm in the bladder cancer mechano-micro-environment

**DOI:** 10.3389/fgene.2026.1896399

**Published:** 2026-07-23

**Authors:** Xiaofei Dong, Zixuan Chen, Yi Chen, Zheng Xiao, Jingjing Zhang, Yingcun Li, Haifang Du

**Affiliations:** 1 The Second Affiliated Hospital of Guangzhou University of Chinese Medicine, Guangzhou, China; 2 Department of Basic Medical Sciences, Gansu University of Chinese Medicine, Lanzhou, China; 3 Gansu Provincial Key Research Base for Humanities and Social Sciences, Dunhuang Medical Literature Compilation and Application Research Center, Lanzhou, China

**Keywords:** Bladder cancer, mechanical stress, mechanotransduction, metabolic reprogramming, stromal remodeling

## Abstract

Recently managing bladder cancer (BLCA) has been hampered by two stubborn challenges, one is high recurrence rate in non-muscle-invasive tumors (NMIBC), the other is muscle-invasive disease (MIBC) showed limited responsiveness to immune checkpoint inhibitors (ICIs). Tumor micro-environment (TME) is characterized exclusively, while the physical and mechanical forces that actively remodel tumors have been largely overlooked. The bladder, a mechanically dynamic organ that undergoes continuous cycles of filling and voiding, provides an exceptionally instructive model for dissecting tumor biology driven by mechanical stress. In this review, we systematically delineates how mechanical stresses in BLCA, including extracellular matrix (ECM) stiffening, solid stress, fluid shear stress, and cyclic stretch-drive metabolic reprogramming, resulting in enhanced glycolysis, glutamine metabolic remodeling, and lactate accumulation. These metabolic alterations subsequently promote fibroblast activation, collagen deposition, and lysyl oxidase (LOX)-mediated matrix crosslinking via epigenetic mechanisms such as histone lactylation. Building upon these mechanisms, we propose a therapeutic rationale that jointly targets mechanotransduction, aberrant metabolism, and the immunosuppressive micro-environment, and we further discuss the distinctive translational advantages of intravesical instillation for locoregional combinatorial delivery. This review aims to provide a novel conceptual framework for overcoming intravesical chemoresistance and ICI resistance in BLCA.

## 1 Introduction

Bladder cancer (BLCA) ranks as the tenth most common malignancy worldwide, with an estimated 614,298 new cases and 220,596 deaths globally in 2022 (Van Hoogstraten et al., 2023). Approximately 75% of newly diagnosed cases present as non-muscle-invasive bladder cancer (NMIBC). Although patients typically receive transurethral resection of bladder tumor (TURBT) combined with intravesical therapy, the 1-year and 5-year recurrence rates of high-risk NMIBC can reach approximately 15%–61% and 31%–78%, respectively, with progression rates of approximately 0.8%–17% and 0.8%–45% (Yeary et al., 2024). For muscle-invasive bladder cancer (MIBC), neoadjuvant chemotherapy combined with radical cystectomy remains the standard regimen; however, the pathological complete response rate to immune checkpoint inhibitor (ICI) monotherapy is only approximately 24.5%, and although the objective response rate of ICI combined with chemotherapy can be improved to 40.6%, the overall efficacy remains limited (Meng et al., 2025). In addition, the failure rate of intravesical *bacillus* Calmette-Guérin (BCG) therapy reaches 30%–40%, and the complete response rate of ICIs in NMIBC ranges from 8% to 41% across various studies, indicating that current therapeutic strategies still face significant clinical bottlenecks (Nishimu et al., 2024).

Research on the BLCA tumor microenvironment (TME) has long focused on biochemical signals. These signals include growth factors, cytokines, and chemokines. However,the bladder tumor progression involves more than just rewiring of signaling networks, also involves mechanical remodeling, which affecting epithelial cells, stromal cells, and the extracellular matrix (ECM) (Shen et al., 2023). A recent study in *Nature Physics* has revealed complex mechanical instabilities in the basement membrane during the early stages of bladder cancer, which implying that mechanical factors may play an initiating role in the development of BLCA (Lampart et al., 2025). Despite large amounts of reviews on mechanotransduction and metabolic regulation in tumors are gradually emerging, few reviews focus specifically on bladder cancer, even though illustrating the relationships between the prevalence of BLCA with mechanical stress, metabolic reprogramming, and matrix remodeling (Metwally et al., 2026).

**FIGURE 1:**
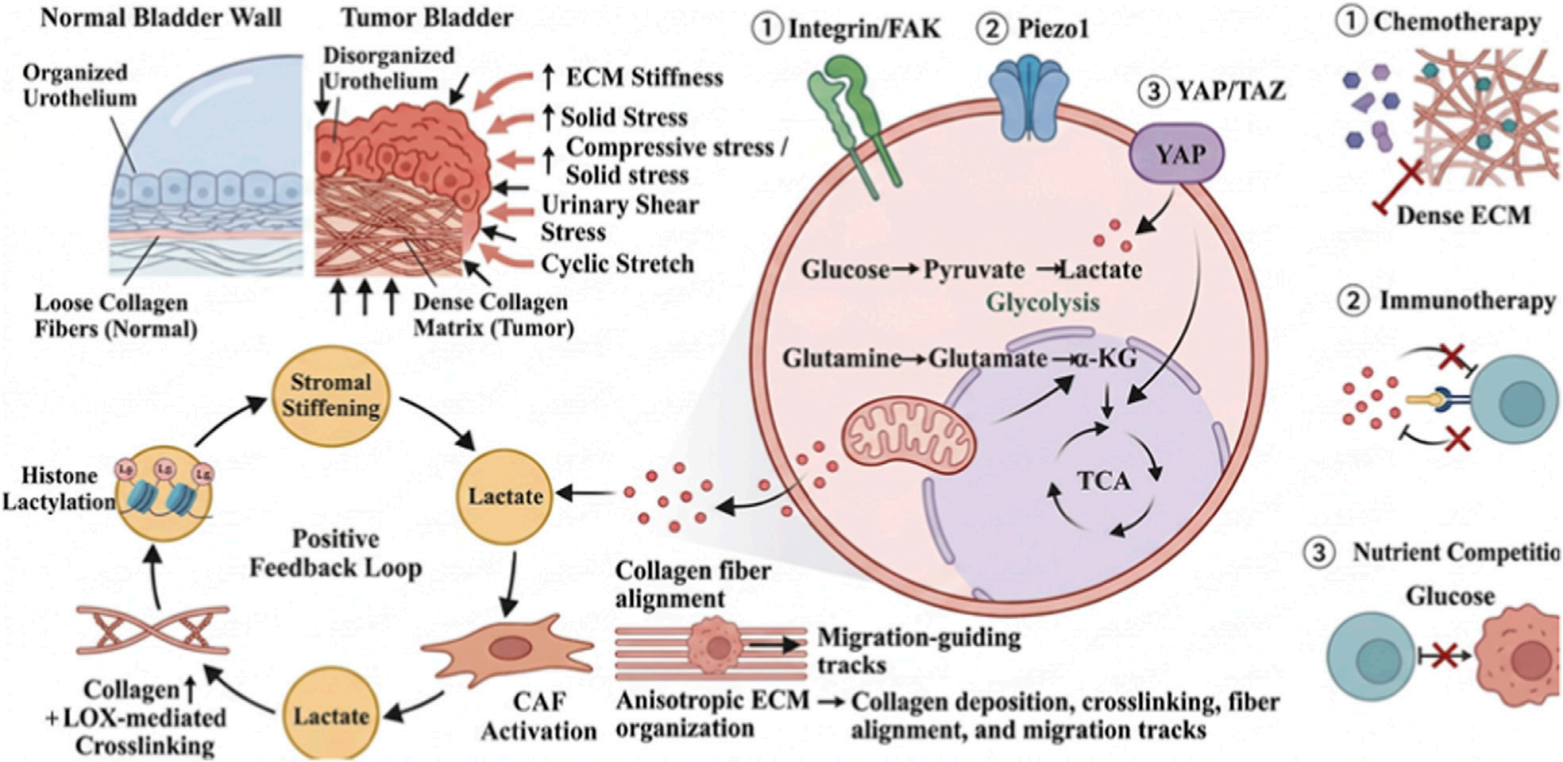
Schematic diagram of the integrated mechanisms linking mechanical stress, metabolic reprogramming, and stromal remodeling in bladder cancer. Significant differences in mechanical properties exist between the normal bladder wall and tumor tissue. In tumor tissue, ECM stiffness, solid stress, compressive stress, and IFP are increased, while dynamic mechanical stimuli, including urinary shear stress and cyclic stretch, continuously act on bladder cancer cells and stromal cells. Mechanosensors such as integrin/FAK, Piezo1, and YAP/TAZ convert extracellular mechanical cues into intracellular signaling events, thereby promoting enhanced glycolysis, glutamine metabolic remodeling, and lactate accumulation. Lactate functions as a metabolic product, signaling molecule, and substrate for epigenetic modification, promoting CAF activation, collagen expression, and LOX-mediated matrix crosslinking via histone lactylation. In addition to collagen deposition and matrix densification, stromal remodeling also involves collagen fiber alignment, anisotropic organization, and the formation of migration-guiding tracks, which facilitate mechanotransduction, cell invasion, and tumor dissemination. These processes establish a positive feedback loop in which stromal stiffening and metabolic aberrations are mutually amplified. This loop attenuates the efficacy of chemotherapy and immunotherapy through physical barriers, nutrient competition, and metabolic immunosuppression. Abbreviations: ECM, extracellular matrix; CAF, cancer-associated fibroblast; LOX, lysyl oxidase; α-KG, α-ketoglutarate; IFP, interstitial fluid pressure; FAK, focal adhesion kinase; YAP/TAZ, Yes-associated protein/transcriptional coactivator with PDZ-binding motif; ICI, immune checkpoint inhibitor.

Owing to the cyclical filling and voiding of the bladder, the urothelium is persistently subjected to urinary shear stress, wall tension, and hydrostatic pressure fluctuations. This distinctive mechanical milieu positions the bladder urothelium as an intrinsically responsive substrate for probing the coupling between mechanical signaling and cellular metabolism (Merrill et al., 2016). The central thesis of this review is that mechanical stress initiates stromal remodeling in BLCA by triggering metabolic reprogramming. This metabolic rewiring establishes a self-reinforcing feedback loop: matrix stiffening drives aberrant metabolic shifts, whose byproducts in turn accelerate ECM deposition and crosslinking to further harden the matrix. An integrated overview of the proposed mechano-metabolic-stromal interaction framework in BLCA is presented in Figure 1. Ultimately, this reciprocal mechano-metabolic coupling may represent a core mechanism underlying both intravesical chemoresistance and resistance to immune checkpoint inhibitors.

## 2 Mechanical stress in the bladder cancer micro-environment: sources and clinical implications

### 2.1 Multidimensional sources of mechanical stress

Several mechanical stressors of pathological relevance operate within the BLCA TME. A primary one is heightened ECM stiffness. Whereas the stiffness of normal bladder wall ECM lies around 2–5 kPa, tumor tissue exhibits a several-fold increase, attributable to excessive collagen accumulation and augmented crosslinking mediated by the lysyl oxidase (LOX) family (Zhou et al., 2026). Three-dimensional models of varying stiffness constructed using collagen and hyaluronic acid hydrogels have demonstrated that increased matrix stiffness can directly maintain bladder cancer stem cell properties and promote tumor recurrence (Metwally et al., 2025).

Another important mechanical abnormality is the elevation of solid stress, compressive stress, and interstitial fluid pressure (IFP). Within the physically constrained tumor microenvironment, rapid neoplastic growth generates residual solid stress and compressive forces that mechanically deform stromal elements, blood vessels, and lymphatic structures, thereby driving a significant increase in IFP. While normal tissue IFP generally remains within the range of 1–3 mmHg, IFP in solid tumors can be elevated by an order of magnitude (Amparo et al., 2025). Such pressure abnormalities not only affect local perfusion but also restrict drug delivery into the deep tumor parenchyma.

Thirdly, the specialized physiology of the bladder exposes its urothelium to uninterrupted fluid shear stress and cyclic mechanical stretch. Shear stress arises from urinary flow, and cyclic distension is produced by repetitive filling and voiding cycles; together, these forces impose a constantly shifting mechanical environment upon urothelial cells. Piezo1, a mechanosensitive cation channel of central importance, transduces these mechanical stimuli by gating stretch-activated Ca^2+^ influx and promoting ATP release in the urothelium (Ma et al., 2025).

### 2.2 Mechanical barriers and clinical imaging assessment

ECM stiffening and elevated IFP undermine therapeutic efficacy by imposing dual physical barriers. A densely crosslinked ECM impedes immune cell infiltration, trapping CD8^+^ T cells at the stromal periphery and thereby fostering an immune-excluded phenotype (Liu et al., 2020). Concurrently, elevated IFP weakens convective drug delivery from vasculature to tumor parenchyma by reducing the transcapillary pressure. Therefore, mechanical abnormalities are not a consequence of tumor progression but directly contribute to the establishment of therapeutic resistance.

In terms of clinical evaluation, shear wave elastography (SWE) can be employed for preoperative assessment of tissue stiffness. Several studies have shown that SWE measurements correlate significantly with histopathological grade, tumor stage, and microvessel density, suggesting potential utility as an imaging biomarker for clinical decision-making (Pisotskyi et al., 2024). Furthermore, the high expression of integrin family members and YAP/TAZ in BLCA is closely associated with adverse prognosis and malignancy (Li et al., 2023).

## 3 Molecular mechanisms by which mechanical stress drives metabolic reprogramming and stromal remodeling

### 3.1 Core mechanosensors: biochemical transduction hubs of mechanical signals

BLCA cells sense and transduce mechanical stress primarily through the Integrin/FAK pathway, Piezo1 channels, and the YAP/TAZ pathway which engage in extensive crosstalk, establishing a first-tier signaling hub that couples mechanical inputs to metabolic reprogramming.

The Integrin/FAK/PI3K/AKT axis represents the classical mechanism by which ECM stiffness signals are transmitted. Besides ECM stiffening enhances integrin clustering and FAK phosphorylation, thereby activating the PI3K/AKT signaling cascade. And the expression of FAK is markedly higher in bladder cancer tissues than in normal tissues, and its inhibition attenuates the proliferative and migratory capacity of BLCA cells (Chi et al., 2026). Upon activation, AKT upregulates glucose transporter 1 (GLUT1), hexokinase 2 (HK2), 6-phosphofructo-2-kinase/fructose-2,6-bisphosphatase 3 (PFKFB3), and lactate dehydrogenase A (LDHA) by stabilizing hypoxia-inducible factor-1α (HIF-1α) and enhancing c-Myc transcriptional activity. Transforming acidic coiled-coil-containing protein 3 (TACC3) has been shown to stabilize c-Myc protein by interacting with c-Myc and promoting its acetylation at K323, thereby enhancing glycolytic flux in BLCA cells (Lin et al., 2025). These findings underscore the pivotal role of the Integrin/FAK downstream in BLCA metabolic reprogramming.

The mechanogated Piezo1 channel serves as a critical sensor of bladder distension. The Piezo channel family is broadly expressed in urinary system and responds to mechanical stimuli including shear stress, bladder wall stretch, and urinary flow (Chen et al., 2023). In bladder urothelium, Piezo1 senses cellular stretch and mediates Ca^2+^ influx and ATP release. Moreover, Piezo1 is involved in integrating mechanical signals and metabolic states in immune cells, suggesting its potential function in TME immunoregulation. Piezo1 blockade also reduces YAP nuclear localization and inhibits the proliferation of bladder urothelial stem cells.

Serving as a master integrator, the YAP/TAZ pathway bridges mechanical signaling and metabolic rewiring. YAP1 overexpression in bladder cancer is associated with tumorigenesis, progression, resistance to cisplatin, and recurrence. Pan-cancer studies further reveal that activated YAP/TAZ transcriptionally upregulates key glycolytic enzymes, thereby driving lactate generation (Liu et al., 2026). Beyond glycolytic regulation, YAP/TAZ also participates in the transcriptional control of genes related to nucleotide and amino acid metabolism, and drives cancer-associated fibroblast (CAF) activation and ECM synthesis by regulating α-smooth muscle actin (α-SMA), connective tissue growth factor (CTGF), collagen, and LOX gene expression. YAP/TAZ can therefore be regarded as a key transcriptional hub linking signals, metabolic states, and stromal remodeling.

Significant crosstalk operates among these mechanosensors. FAK can regulate the Hippo pathway, facilitating YAP/TAZ nuclear localization, while Piezo1-dependent Ca^2+^ influx increases cytoskeletal tension and promotes YAP nuclear translocation via RhoA/ROCK signaling. YAP, reciprocally, feedback-controls the transcription of integrin and FAK genes. Because the bladder undergoes continuous cycles of filling and voiding, the urothelium is persistently exposed to changing mechanical forces. This unique physiological setting creates an environment permissive to the chronic activation of these mechanosensory cascades.

### 3.2 Mechanical stress-driven metabolic reprogramming

#### 3.2.1 Mechanical remodeling of glycolysis and lactate metabolism

In bladder cancer, HK2, pyruvate kinase M2 (PKM2), and LDHA are aberrantly expressed (Pan et al., 2026). Matrix stiffness and mechanical stress can upregulate HK2, PFKFB3, and LDHA via the YAP/TAZ and FAK signaling pathways in pan-cancer. In chemoresistant BLCA cells, STAT3 activation-mediated overexpression of HK2 and LDHA enhances glycolytic flux and further promotes the transdifferentiation of normal fibroblasts into CXCL14-secreting CAFs, thereby establishing a self-reinforcing feedback loop that sustains chemoresistance (Xiang et al., 2025).

Notably, researchers investigated the relationship between the mechanical properties of bladder cells and glycolytic activity with atomic force microscopy (AFM), demonstrating that the mechanical stiffness of bladder cancer cells correlates positively with glycolytic activity and lactate levels (Zhu et al., 2023). This finding provides early experimental evidence that alterations in cellular stiffness are interrelated with changes in metabolic activity in BLCA. Although the study did not fully resolve the upstream mechanotransduction pathways, it suggested a direct link among cytoskeletal state, cellular mechanical properties, and glycolytic metabolism, providing important support for subsequent investigations of mechanical stress-driven metabolic reprogramming. In addition, Kim et al. observed that compressive stress can induce upregulation of glycolytic genes such as ENO2, HK2, and PFKFB3 in CAFs and increase lactate production (Kim et al., 2019), further indicating that mechanical loading can directly regulate stromal cell metabolism.

Lactate possesses multiple biological identities within the mechanically regulated TME. Lactate is exported via monocarboxylate transporter 4 (MCT4) to sustain nicotinamide adenine dinucleotide (NAD^+^) regeneration and intracellular acid-base homeostasis. As a signaling molecule, lactate mediates autocrine or paracrine signaling through G protein-coupled receptor 81 (GPR81); in bladder cancer, tumor-derived lactate can promote malignant progression and chemoresistance via STAT3-related pathways (Kim et al., 2019; Silva et al., 2025). More importantly, lactate also serves as a substrate for epigenetic modifications, regulating gene expression through histone lysine lactylation (Xu et al., 2025), which establishes a direct molecular link between metabolic reprogramming and stromal remodeling.

#### 3.2.2 Amino acid metabolism and metabolic symbiosis

Beyond glycolysis, mechanical signaling also modulates amino acid metabolism. In tumor, YAP/TAZ and c-Myc-associated signaling pathway upregulates glutaminase 1 (GLS1), thereby driving glutaminolysis (Yang et al., 2024). And, α-Ketoglutarate (α-KG) generated from glutamine metabolism functions not as a tricarboxylic acid (TCA) cycle intermediate but as an essential co-factor for ten-eleven translocation (TET) and Jumonji C domain-containing (JmjC) family demethylases, thereby directly coupling glutamine metabolism with histone demethylation (Yi et al., 2021). This process suggests that YAP/TAZ and c-Myc-associated signaling pathway may alter chromatin states via metabolic intermediates, thereby further influencing the phenotypes of tumor.

Then metabolic symbiosis further deepens the coupling suffering mechanical stress, metabolic reprogramming, and stromal remodeling. Cancer cells secrete lactate via aerobic glycolysis, which CAFs uptake through monocarboxylate transporter 1 (MCT1) and utilize for oxidative phosphorylation, becoming activated toward a pro-tumorigenic phenotype, termed the reverse Warburg effects (Hsu et al., 2026). Activated CAFs secrete ECM components, reinforcing the matrix structure, above all, metabolic symbiosis enables the spatial diffusion and sustained amplification of local mechanical signals within the TME.

### 3.3 Metabolic reprogramming drives stromal remodeling and establishes a positive feedback loop

#### 3.3.1 CAF activation and ECM remodeling

CAFs in BLCA display marked heterogeneity. Single-cell transcriptomic analyses have identified multiple CAF subtypes within bladder urothelial carcinoma, including myofibroblast-like CAFs, inflammatory CAFs, and other mixed features (Zhang et al., 2024). These CAF subtypes show differential enrichment and collectively participate in ECM deposition, inflammatory cytokine secretion, and immune modulation.

Mechanical stress-induced glycolysis and lactate accumulation contributes to CAF activation. Notably, Bhatt et al. demonstrated that lactate directly upregulates the expression of COL1A1, COL1A2, and LOX genes in CAFs via histone lactylation, thereby driving matrix remodeling in a pancreatic cancer model (Vera et al., 2025), which constitutes a relatively complete causal chain extending from mechanical stimulation to metabolite accumulation, and further to epigenetic modification and enhanced stromal synthesis. Although it requires further validation in BLCA models, offering how metabolic reprogramming drives stromal remodeling in bladder cancer.

LOX- and LOXL2-mediated collagen crosslinking further bridges metabolic states with ECM mechanical properties. LOX family members catalyze the crosslinking of collagen and elastin, converting soluble collagen into insoluble mature fibers and thereby increasing matrix stiffness (Li et al., 2018). In urological tumors and fibrosis-related pathologies, LOX family members are regarded as important regulators of ECM remodeling (Li et al., 2018). This implies that glucose and glutamine metabolism not only influence intracellular signaling and epigenetic states via lactate and α-KG but may also alter tissue-level mechanical properties through effects on collagen synthesis, crosslinking enzyme activity, and matrix deposition. Importantly, stromal remodeling involves not only increased collagen abundance and matrix densification but also changes in collagen architecture, including fiber alignment, anisotropic organization, and the formation of migration-guiding tracks, which can further enhance mechanotransduction, cell invasion, and tumor dissemination.

#### 3.3.2 Positive feedback loop and immunosuppression

The above mechanisms collectively constitute a self-reinforcing vicious cycle: ECM stiffening activates mechanosensors, which in turn drive glycolytic and glutamine metabolic remodeling via FAK, Piezo1, and YAP/TAZ pathways; the metabolic product lactate further promotes CAF activation, collagen deposition, and LOX-mediated matrix crosslinking through signal transduction and histone lactylation, ultimately leading to further ECM stiffening. Once established, this cycle drives the TME into a persistently reinforced state of mechanical and metabolic aberration.

This feedback loop also offers a multi-level explanation for the limited efficacy of ICIs and chemotherapy in bladder cancer. At the physical level, a dense and highly crosslinked ECM forms a barrier that prevents CD8^+^ T cells from infiltrating the tumor parenchyma, restricting them to the stromal periphery and producing an immune-excluded phenotype. At the metabolic level, tumor cells and CAFs upregulate glucose and amino acid transporters, allowing them to outcompete T cells for glucose, glutamine, and other nutrients, thereby limiting the metabolic resources essential for T-cell activation and proliferation (Swamy et al., 2016). In addition, high levels of lactate can stabilize programmed death-ligand 1 (PD-L1) protein through lactylation modification, thereby promoting tumor immune evasion (Liang et al., 2026); meanwhile lactate accumulation also suppresses T-cell and NK-cell function and induces polarization of immunosuppressive cells (Brand et al., 2016). Hence, physical barriers, nutrient competition, and metabolic immunosuppression synergistically constitute a critical microenvironmental basis for therapeutic resistance in BLCA. To provide a concise overview of the complex interactions, the major mechanical stress stimuli, corresponding mechanosensors and signaling pathways, metabolic reprogramming events, stromal remodeling outcomes, and key supporting references are summarized in Table 1.

**TABLE 1:** Summary of mechanical stress-driven metabolic reprogramming and stromal remodeling in BLCA.

Mechanical stress stimulus	Principal mechanosensors and signaling pathways	Metabolic reprogramming events	Stromal remodeling outcomes	Ref.
ECM stiffening and increased matrix rigidity	Integrin/FAK/PI3K/AKT signaling; YAP/TAZ activation	Enhanced glycolysis; upregulation of GLUT1, HK2, PFKFB3, and LDHA; lactate accumulation	CAF activation; collagen deposition; LOX-mediated matrix crosslinking; further ECM stiffening	Zhou et al. (2026), Metwally et al. (2025), Chi et al. (2026), Lin et al. (2025), Liu et al. (2026), Pan et al. (2026), Zhu et al. (2023), Vera et al. (2025), Li et al. (2018)
Solid stress and elevated IFP	Compression-associated cytoskeletal signaling; FAK- and YAP/TAZ-related mechanotransduction	Upregulation of glycolytic genes such as ENO2, HK2, and PFKFB3; increased lactate production	Stromal compression; impaired perfusion and drug penetration; reinforcement of a therapy-resistant TME	Amparo et al. (2025), Ma et al. (2025), Chi et al. (2026), Liu et al. (2026), Kim et al. (2019)
Urinary shear stress and cyclic stretch	Piezo1-mediated Ca^2+^ influx; ATP release; RhoA/ROCK-related cytoskeletal remodeling; YAP nuclear translocation	Ca^2+^-dependent signaling activation; potential coupling with glycolytic and glutamine-related metabolic adaptation	Urothelial activation; enhanced tumor-stromal communication; promotion of proliferative and remodeling phenotypes	Merrill et al. (2016), Ma et al. (2025), Chen et al. (2023), Liu et al. (2026), Yang et al. (2024)
Chronic mechanosensor crosstalk	Integrin/FAK, Piezo1, and YAP/TAZ feedback regulation	Sustained activation of glycolytic and amino acid metabolic programs	Persistent mechano-metabolic activation and stromal remodeling	Ma et al. (2025), Chi et al. (2026), Liu et al. (2026)
Lactate accumulation under mechanical and metabolic stress	MCT4-mediated lactate export; GPR81 signaling; STAT3-related pathways; histone lactylation	Lactate functions as a metabolic product, signaling molecule, and epigenetic substrate	CAF activation; collagen and LOX-related gene expression; ECM deposition; immunosuppressive remodeling	Kim et al. (2019), Silva et al. (2025), Xu et al. (2025), Vera et al. (2025), Liang et al. (2026), Brand et al. (2016)
Glutamine metabolic remodeling and metabolic symbiosis	YAP/TAZ- and c-Myc-associated signaling; GLS1 activation; MCT1-mediated lactate uptake by CAFs	Enhanced glutaminolysis; α-KG generation; reverse Warburg-like metabolic coupling between cancer cells and CAFs	CAF metabolic activation; ECM synthesis; amplification of local mechanical and metabolic signals	Yang et al. (2024), Yi et al. (2021), Hsu et al. (2026)
LOX/LOXL2-mediated matrix crosslinking	LOX-family enzymes; stiffness-induced integrin/FAK and YAP/TAZ signaling	Reinforcement of stiffness-driven metabolic rewiring	Progressive collagen crosslinking; increased matrix stiffness; immune exclusion; impaired therapeutic delivery	Chi et al. (2026), Liu et al. (2026), Vera et al. (2025), Li et al. (2018)

## 4 Therapeutic strategies targeting mechanosensing, metabolic aberrations, and stromal remodeling

### 4.1 Current therapeutic strategies and unmet clinical needs in BLCA

Current BLCA management is largely stage-dependent, yet each major therapeutic modality remains constrained by specific clinical limitations. These strategies and their principal bottlenecks are summarized in Table 2. In NMIBC, high recurrence rates, limited intravesical drug retention and penetration, and BCG failure remain major clinical challenges. In MIBC and advanced disease, heterogeneous responses to chemotherapy, morbidity associated with radical cystectomy, and suboptimal responses to immune checkpoint inhibitors continue to limit durable disease control. These unmet needs underscore the rationale for therapeutic strategies that extend beyond tumor cell-intrinsic targets and instead address the physical, metabolic, and stromal features of the BLCA microenvironment.

**TABLE 2:** Summary of current therapeutic strategies and major limitations in bladder cancer.

Clinical setting	Current therapeutic strategy	Main therapeutic rationale	Major limitations	Ref.
Non-muscle-invasive bladder cancer (NMIBC)	Transurethral resection of bladder tumor (TURBT) with or without intravesical chemotherapy	Removes visible tumors and reduces local recurrence risk	High recurrence rate, incomplete eradication of residual tumor cells, limited drug retention and penetration, and frequent need for repeated interventions	Yeary et al. (2024), Nishimu et al. (2024)
Intermediate- and high-risk NMIBC	Intravesical *Bacillus* Calmette-Guérin (BCG) immunotherapy	Induces local immune activation within the bladder mucosa	BCG failure or intolerance remains common; approximately 30%–40% of patients experience treatment failure; immune-excluded and metabolically suppressive tumor microenvironment may weaken efficacy	Nishimu et al. (2024)
Muscle-invasive bladder cancer (MIBC)	Neoadjuvant chemotherapy followed by radical cystectomy	Provides systemic cytotoxic control and improves surgical outcomes in selected patients	Heterogeneous treatment response, chemotherapy resistance, systemic toxicity, and substantial impact on urinary function and quality of life	Meng et al. (2025)
Advanced or metastatic bladder cancer	Immune checkpoint inhibitor (ICI) monotherapy	Restores antitumor T-cell activity by blocking immune checkpoint signaling	Response rates remain limited in many patients; stromal barriers, immune exclusion, nutrient competition, and lactate accumulation may contribute to resistance	Meng et al. (2025), Nishimu et al. (2024)

### 4.2 Targeting mechanosensors

The Piezo1-specific inhibitor GsMTx4 reversibly inhibits stress-induced Ca^2+^ influx by blocking mechanosensitive cation channels and attenuating downstream YAP nuclear translocation and activation. Dooku1, a selective small-molecule antagonist of Piezo1, is also undergoing evaluation in preclinical models. It should be noted that direct evidence supporting Piezo1 inhibition in BLCA remains relatively limited, and more systematic bladder cancer-specific studies are needed in the future to confirm its antitumor activity and safety.

With respect to the YAP/TAZ pathway, Verteporfin has been identified as a small-molecule compound that disrupts the transcriptional enhanced associate domain (TEAD)-YAP interaction (Song et al., 2016). Among next-generation TEAD inhibitors, IAG933, a small-molecule selective disruptor of the YAP/TEAD protein-protein interaction, has entered phase I clinical trials (Chapeau et al., 2024), and phase I/II clinical results for VT3989, an oral inhibitor of TEAD palmitoylation, have also been reported (Yap et al., 2025). With regard to the Integrin/FAK pathway, Defactinib (VS-6063), a FAK inhibitor, has completed phase II evaluation in the EAY131-U subprotocol of the National Cancer Institute Molecular Analysis for Therapy Choice (NCI-MATCH) trial for neurofibromin 2 (NF2)-deficient solid tumors, although its single-agent activity was limited (Spallarossa et al., 2022). These results suggest that single-agent blockade of mechanosensors may be insufficient to fully reverse the complex TME state, and that a more rational direction is combination with metabolic targeting, stromal normalization, or immunotherapy.

### 4.3 Targeting mechanically induced metabolic vulnerabilities

Targeting the lactate axis primarily involves inhibiting lactate generation and blocking lactate transport. AZD3965, a selective MCT1 inhibitor, blocks lactate efflux, leading to intracellular accumulation of lactate and protons in tumor cells and thereby disrupting metabolic homeostasis in glycolysis-dependent cells. The results of its first-in-human phase I dose-escalation trial have been reported (Beloueche-Babari et al., 2020). Telaglenastat (CB-839), a selective oral GLS1 inhibitor, serves as a prototypical compound for targeting glutamine metabolism by suppressing glutaminolysis. Its combination with the immune checkpoint inhibitor nivolumab has undergone phase I/II safety evaluation in cohorts encompassing metastatic melanoma, renal cell carcinoma, and non-small-cell lung cancer. While the regimen was deemed tolerable, clinical activity varied substantially across tumor types and was ultimately constrained in magnitude (Gouda et al., 2025). The researches indicate that the clinical metabolic inhibitors requires more precise patient stratification.

PFKFB3, a key regulatory protein of glycolysis, is similarly a potential metabolic intervention target. The molecular inhibitor 3PO, first described by Clem, targets PFKFB3 activity, resulting in diminished glucose uptake, lactate production, ATP levels, and glycolytic flux, while also suppressing tumor growth *in vivo*. These observations provided critical experimental evidence that pharmacological inhibition of PFKFB3 can effectively attenuate the glycolytic dependence of tumors (Imbert-Fernandez et al., 2024). However, direct efficacy validation of PFKFB3 inhibition in BLCA remains insufficient, and its translational value warrants further evaluation using bladder cancer mechanical models, organoids, and animal models.

### 4.4 Stromal normalization and locoregional combination therapeutic strategies

Efforts to normalize the ECM aim to attenuate collagen crosslinking, decrease matrix rigidity, and facilitate drug penetration. Simtuzumab, an anti-LOXL2 monoclonal antibody, has been assessed in multiple phase II clinical trials for pancreatic cancer, colorectal cancer, and idiopathic pulmonary fibrosis. Nevertheless, the agent has generally failed to demonstrate conclusive evidence of clinical efficacy (Su et al., 2024). Such results imply that single-target inhibition of LOXL2 is likely insufficient, given the compensatory activities among LOX family members. PXS-5505, a next-generation pan-LOX inhibitor, concurrently suppresses LOX and LOXL isoforms. In models of pancreatic cancer, it has been shown to dampen CAF-mediated collagen matrix remodeling and crosslinking, thereby enhancing tumor sensitivity to gemcitabine (Chitty et al., 2023). And Pegvorhyaluronidase alfa (PEGPH20) reduces IFP by degrading hyaluronan components of the ECM, thereby improving drug delivery and immune cell infiltration (Chiorean et al., 2026).

The bladder constitutes an advantageous site for intracavitary drug delivery, as transurethral catheterization permits direct administration of therapeutic agents into the bladder lumen. This approach generates high local drug concentrations at the tumor site while limiting systemic exposure. In light of the mechanisms delineated earlier, we propose a locoregionally integrated combination regimen encompassing mechanical decompression, metabolic reprogramming, and immunotherapy. Specifically, stromal-relaxing agents could be utilized to diminish ECM crosslinking and interstitial fluid pressure, thus enhancing drug penetration and immune cell trafficking. Metabolic inhibitors targeting MCT1, GLS1, or PFKFB3 could concurrently suppress lactate accumulation, glutaminolysis, and glycolytic flux. Following partial alleviation of the physical barriers and metabolically imposed immunosuppression, immune checkpoint inhibitors would be expected to more potently evoke CD8^+^ T cell-mediated anti-tumor activity. While this combinatorial paradigm presently remains conceptual and preclinical, the delivery advantages conferred by intravesical instillation provide a strong translational rationale for further exploration. Representative candidate agents targeting mechanosensing, metabolic vulnerabilities, and stromal remodeling are summarized in Table 3.

**TABLE 3:** Representative candidate agents targeting the mechano-metabolic-stromal axis in bladder cancer.

Targeting dimension	Representative agent	Core target	Principal mechanism	Ref.
Mechanosensor blockade	GsMTx4/Dooku1	Piezo1	Inhibits mechanical stress-induced Ca^2+^ influx, attenuating downstream YAP nuclear localization	Ma et al. (2025), Chen et al. (2023)
	Verteporfin	YAP/TEAD	Disrupts the YAP-TEAD interaction and inhibits YAP-dependent transcription	Song et al. (2016)
	IAG933	YAP/TEAD	Selectively disrupts the YAP/TEAD protein-protein interaction	Chapeau et al. (2024)
	VT3989	TEAD	Inhibits TEAD palmitoylation, blocking YAP/TAZ transcriptional output	Yap et al. (2025)
	Defactinib (VS-6063)	FAK	Inhibits FAK, blocking integrin-downstream signaling	Spallarossa et al. (2022)
Metabolic targeting	AZD3965	MCT1	Blocks lactate transport, inducing intracellular acidification	Beloueche-Babari et al. (2020)
	Telaglenastat (CB-839)	GLS1	Inhibits glutaminolysis, attenuating α-KG generation	Gouda et al. (2025)
	3PO	PFKFB3	Reduces glucose uptake, lactate production, and glycolytic flux	Imbert-Fernandez et al. (2024)
Stromal normalization	Simtuzumab	LOXL2	Inhibits LOXL2-mediated collagen crosslinking (limited monotherapy benefit)	Su et al. (2024)
	PXS-5505	LOX/LOXL family	Inhibits LOX-family enzymes, reducing crosslinking and softening matrix	Chitty et al. (2023)
	PEGPH20	Hyaluronan	Degrades hyaluronan, reducing IFP and improving drug delivery	Chiorean et al. (2026)

Going forward, a multimodal biomarker framework should guide patient selection and efficacy assessment. SWE enables the serial, quantitative monitoring of tissue stiffness, while circulating ECM fragments and LOX activity levels could functioned as accessible pharmacodynamic readouts of stromal remodeling; and urinary or tissue metabolomic indices can reflect local metabolic states. Combined stratification using mechanical imaging, stromal biomarkers, and metabolomics is expected to enhance the precision of triple combination therapy strategies.

## 5 Conclusions and perspectives

In this review, we systematically establish that mechanical stress metabolic reprogramming and the resultant stromal remodeling play a critical role in shaping the BLCA TME. Increased ECM stiffness, solid stress, and dynamic hydrodynamic forces-are relayed through the Integrin/FAK, Piezo1, and YAP/TAZ axis, triggering a metabolic shift characterized by elevated glycolysis, lactate accumulation, and glutamine-centered metabolic rewiring. The resultant metabolites, most notably lactate, foster CAF activation and ECM deposition via epigenetic routes, while LOX-facilitated collagen crosslinking further consolidates matrix stiffening. This positive feedback loop, perpetuated by physical exclusion, nutrient deprivation, and metabolically imposed immunosuppression, emerges as a plausible unifying mechanism underlying the clinical conundrums of intravesical chemoresistance and poor response to immune checkpoint inhibitors in BLCA.

The bladder not only provides a unique model for investigating the coupling between mechanical signaling and metabolism but also offers a natural delivery conduit for combination therapy. In the near term, translational research may prioritize agents with existing clinical or preclinical foundations, such as Verteporfin, Defactinib, Telaglenastat, AZD3965, 3PO-related PFKFB3 inhibitory strategies, PXS-5505, and PEGPH20, and evaluate their safety and pharmacodynamics in intravesical instillation or in combination with ICIs for BLCA. In the medium to long term, bladder cancer organoids, microfluidic organ-on-chip systems, spatial mechano-omics, and multimodal AI-based analyses will facilitate reconstruction of the dynamic interactions among tumor cells, immune cells, stroma, and mechanical forces (Kang et al., 2025). An integrated therapeutic approach targeting mechanosensing, metabolic aberrations, and stromal remodeling holds promise for providing novel strategies to overcome intravesical chemoresistance and immune resistance in BLCA.
